# Cigarette smoking exposure and clinical outcomes in Graves' disease

**DOI:** 10.1210/clinem/dgag061

**Published:** 2026-02-12

**Authors:** Sarah Forbes, May Loo, Su Ann Tee, Kathryn Stewart, Jonathan Vernazza, Lauren Carroll, Nicholas Vennart, Peter Bartholomew, Kilimangalam Narayanan, Vasileios Tsatlidis, Salman Razvi

**Affiliations:** Department of Endocrinology, Gateshead Health NHS Foundation Trust, Gateshead NE9 6SX, UK; Department of Endocrinology, Gateshead Health NHS Foundation Trust, Gateshead NE9 6SX, UK; Department of Endocrinology, Gateshead Health NHS Foundation Trust, Gateshead NE9 6SX, UK; Department of Endocrinology, Gateshead Health NHS Foundation Trust, Gateshead NE9 6SX, UK; Department of Biochemistry, South of Tyne Pathology Services, Gateshead Health NHS Foundation Trust, Gateshead NE9 6SX, UK; Department of Biochemistry, South of Tyne Pathology Services, Gateshead Health NHS Foundation Trust, Gateshead NE9 6SX, UK; Department of Medical Physics, South Tyneside and Sunderland NHS Foundation Trust, Sunderland SR4 7TP, UK; Department of Medical Physics, South Tyneside and Sunderland NHS Foundation Trust, Sunderland SR4 7TP, UK; Department of Endocrinology, Gateshead Health NHS Foundation Trust, Gateshead NE9 6SX, UK; Department of Endocrinology, Gateshead Health NHS Foundation Trust, Gateshead NE9 6SX, UK; Department of Endocrinology, Gateshead Health NHS Foundation Trust, Gateshead NE9 6SX, UK; Translational and Clinical Research Institute, Centre for Life, Newcastle University, Central Parkway, Newcastle upon Tyne NE1 3BZ, UK

**Keywords:** Graves' disease, cigarette smoking, TRAbs, relapse, orbitopathy, severity, dose–response

## Abstract

**Context:**

Smoking is a recognized risk factor for Graves' disease (GD), but its quantitative relationship with disease severity, autoimmunity, and relapse after antithyroid drug (ATD) therapy is unclear.

**Objective:**

To evaluate the dose-dependent association between smoking exposure and key clinical outcomes in GD.

**Methods:**

This was a single-center observational cohort study conducted in a secondary-care endocrinology unit in the United Kingdom. Consecutive adults with newly diagnosed GD confirmed by suppressed TSH, elevated thyroid hormones, and positive TRAb or diffusely increased scintigraphic uptake were analysed. All 991 eligible patients were included in baseline analyses; 663 completed ≥12 months follow-up after ATD cessation, and 712 contributed to long-term relapse analyses. The main outcome measures were baseline TRAb levels, presence of orbitopathy, and relapse at 12 months and long term, defined as recurrent biochemical hyperthyroidism with elevated TRAb after ATD withdrawal. Other outcomes included additional biochemical, scintigraphic and clinical measures. Exposures were smoking status (non-, ex-, current smoker) and cigarettes/day.

**Results:**

Current smokers (27%) had higher TRAb levels at diagnosis (median 7.8 vs 6.6 IU/L in nonsmokers, *P* = .002) and increased odds of orbitopathy (OR 1.76, 95% CI 1.17-2.64). Each 10 cigarettes/day conferred a 34% (95% CI 10-79%) higher odds of orbitopathy and 60% higher 12-month relapse risk. Smokers also had higher TRAb at ATD cessation. In time-dependent Cox models, excess relapse risk among current smokers was greatest early after ATD withdrawal (HR 1.24 at 6 months) and diminished by 2 years. TRAb mediated only 7% of the smoking–relapse association.

**Conclusion:**

Smoking is associated with greater autoimmune activity, higher orbitopathy risk, and increased relapse in a dose-dependent manner. Ex-smokers have risks comparable with nonsmokers, supporting cessation or reduction as a meaningful intervention to improve GD outcomes.

Graves' disease (GD) is an autoimmune thyroid disorder caused by thyrotropin receptor antibodies (TRAb) that stimulate thyroid hormone production and, in some patients, lead to Graves' orbitopathy (GO) (1, 2). Although genetic susceptibility is central to disease risk, environmental exposures strongly influence disease expression. Among these, cigarette smoking is the most consistent and potent modifiable factor, associated with higher incidence of GD, greater disease severity, and poorer treatment outcomes (3-5).

Epidemiologic studies consistently show that smokers with GD are more likely to develop GO and to relapse after antithyroid drug (ATD) withdrawal, suggesting that smoking aggravates both thyroidal and extrathyroidal autoimmunity (6-8). Several mechanisms plausibly explain these effects. Cigarette smoke promotes oxidative stress and immune activation, enhancing TRAb production (9). In orbital fibroblasts, nicotine and hypoxia stimulate proliferation and adipogenesis, amplifying local autoimmune inflammation (10). These observations provide biologic plausibility for the observed impact of smoking on GD severity.

However, most prior studies have dichotomized exposure as “smoker” vs “nonsmoker,” overlooking potential dose–response relationships and limiting insight into whether risk increases proportionally with smoking intensity (6-8). Quantitative assessment of cigarettes smoked per day may reveal whether even moderate smoking worsens outcomes and whether partial reduction meaningfully attenuates disease activity. Although multiple clinical factors may influence both smoking behavior and disease course (11, 12), few studies have comprehensively adjusted for these variables, leaving uncertainty about whether smoking independently predicts GD severity or relapse.

As novel immunotherapies for GD are under evaluation, these approaches may be costly, complex to administer, and associated with adverse effects (13). In contrast, smoking cessation, or even reduction, represents a simple, low-cost intervention with potential advantages spanning beyond thyroid health for both patients and healthcare systems.

This study therefore aimed to evaluate the dose-dependent association between cigarette smoking and clinical outcomes in a large, real-world GD cohort. Smoking exposure was quantified both categorically (smoking status) and continuously (cigarettes per day), and its relationship with disease severity, TRAb levels, GO, treatment duration, and relapse after ATD therapy was examined. We hypothesized that greater smoking exposure would be associated with higher TRAb titers, increased prevalence of GO, and greater relapse risk independent of other determinants of disease activity.

## Materials and methods

### Study design and participants

This was a retrospective analysis of prospectively collected data from an observational cohort at Gateshead Health NHS Trust, United Kingdom (October 2007-October 2025). Consecutive adults (≥18 years) with newly diagnosed GD were eligible. Diagnosis required biochemical hyperthyroidism (elevated free thyroxine [FT4] and/or free triiodothyronine [FT3] with suppressed TSH) and either positive TRAb or diffusely increased uptake on thyroid scintigraphy.

Patients with prior definitive therapy (radioiodine or thyroidectomy), inactive disease, pregnancy, or immunosuppressive treatment were excluded.

The study was reported in accordance with the strengthening the reporting of observational studies in epidemiology (STROBE) guidelines (14).

### Assessments

At baseline, standardized clinical assessments included the presence of GO, graded as per EUGOGO criteria (15), and calculation of body mass index (BMI). Hyperthyroid symptom burden (palpitations, tremor, weight loss, heat intolerance/sweating, increased gastrointestinal motility, and fatigue) was scored from 0 to 6.

Thyroid function (TSH, FT4, FT3), TRAb and thyroid peroxidase antibodies (TPOAb) measurements were analyzed on the Roche Elecsys electrochemiluminescence immunoassay platform (Cobas e602 until April 2017 and Cobas e801 subsequently). Reference intervals changed marginally over time: TSH 0.4-4.0 to 0.3-4.5 mIU/L, FT4 9-25 to 10-22 pmol/L, FT3 3.5-7.0 to 3.1-6.8 pmol/L, TRAb from <1.0 to <1.8 U/L, and TPOAb from <35 to <34 IU/mL. Results were interpreted using assay-specific reference ranges applicable at the time of measurement.

Between 2007 and 2015, TRAb measurement and technetium (Tc-99m) thyroid scintigraphy were both performed before starting ATD therapy, whereas after 2015, all patients had only TRAb levels measured. Thyroidal area and pertechnetate uptake were measured from planar anterior images (80 MBq 99mTc), using standard ROI-based quantification by 2 blinded specialists.

### Treatment and follow-up

Patients received standard ATD therapy (carbimazole or propylthiouracil) and were reviewed every 3-6 months. Treatment decisions, including transition to radioiodine or surgery, were made according to clinical judgment and patient preference. Antithyroid drug was usually discontinued when TRAb normalized or, in selected cases, based on patient preference or adverse effects. Treatment duration (months) was calculated from therapy initiation to cessation.

### Definition of relapse

Relapse was defined as recurrent biochemical hyperthyroidism (elevated FT4/FT3 with suppressed TSH) and elevated TRAb following ATD withdrawal in patients who had achieved euthyroidism. Time to relapse was measured from ATD cessation to biochemical recurrence. Patients proceeding to definitive therapy before relapse were censored at the time of treatment.

### Assessment of smoking exposure

Smoking status was determined by structured interview at diagnosis and classified as:

- Current smoker: any cigarette use within the previous month
- Ex-smoker: abstinent ≥1 month before diagnosis
- Nonsmoker: no prior regular tobacco use

Quantitative exposure was assessed as self-reported cigarettes smoked per day. For dose–response analyses, this was modeled as a continuous variable. E-cigarette users were categorized as non- or ex-smokers according to prior tobacco use.

### Outcomes

Primary outcomes were:

1. Disease severity at diagnosis (TRAb and thyroid hormone levels)
2. Presence of GO
3. Relapse of hyperthyroidism at 12 months and long-term (up to October 2025) after ATD cessation

Secondary outcomes included symptom score, TPOAb levels, Tc-99m uptake and thyroidal area at diagnosis, treatment duration, and time to relapse.

### Statistical analysis

The study size was determined by the number of consecutive eligible patients with newly diagnosed GD seen at our center during the study period. No formal sample size calculation was performed because this was an observational study using all available data. Continuous variables were summarized as mean ± SD or median (IQR), and categorical variables as counts (%). Between-group comparisons used ANOVA or Kruskal–Wallis tests with Bonferroni-adjusted Dunn's post hoc tests for continuous variables and χ² tests for categorical variables. Missing data were handled by complete-case analysis for each model, with no imputation performed. The proportion of missing data for each variable is reported in the tables. Baseline characteristics of patients with missing vs complete data were similar.

Associations between smoking exposure and baseline outcomes were examined using multivariable linear or logistic regression adjusted for age, sex, BMI, and baseline TRAb (except when TRAb was the dependent variable). Skewed variables (TRAb, TPOAb, FT4, FT3) were log-transformed, and coefficients were exponentiated to yield percentage change. Smoking was analyzed both categorically (smoking status) and continuously (cigarettes/day); nonlinearity was assessed with restricted cubic splines (4 knots) and tested by Wald statistics. To aid interpretability, effects are presented per 10 cigarettes/day.

Differences in ATD treatment duration between smoking groups were analyzed using Kaplan–Meier curves, censoring patients still on therapy. The log-rank test compared time-to-cessation curves. Multivariable Cox regression models adjusted for age, sex, and baseline TRAb were used to estimate hazard ratios.

For patients with TRAb measured at ATD cessation, log-transformed values were regressed on age, sex, smoking status (or cigarettes/day), treatment duration, and baseline log TRAb. Model residuals were assessed for normality and fit.

The association between smoking and symptom score (0-6) was tested using ordinal logistic regression adjusted for age, sex, BMI, and baseline FT4. Associations with GO and 12-month relapse were assessed using multivariable logistic regression, adjusting for age, sex, BMI, TRAb, and ATD duration. Results were expressed as adjusted odds ratios (ORs) with 95% confidence intervals (CIs). Long-term relapse risk was analyzed using Cox proportional hazards models adjusted for age, sex, BMI, baseline TRAb, and ATD duration. Proportional hazards were evaluated using Schoenfeld residuals; where violated, time-dependent Cox and Royston–Parmar spline models were fitted. Patients were censored at the time of definitive therapy (radioiodine or surgery), death, loss to follow-up (having moved away or stopped attending appointments) or end of the follow-up period. Cox models inherently accounted for variable follow-up time and loss to follow-up through right-censoring.

Mediation analysis assessed whether baseline TRAb mediated the association between current smoking and relapse (12-month and long-term). Analyses used the *mediation* package in R with 5000 quasi-Bayesian simulations to estimate average causal mediation (ACME), direct, and total effects.

Statistical significance was defined as 2-tailed *P* < .05. Analyses were conducted in R, version 2025.05.1(R Foundation for Statistical Computing) and SPSS (version 31, IBM Corp, Ill).

### Ethical considerations

This study used routinely collected clinical data from endocrinology outpatients. No interventions or identifiable patient data were included. In accordance with UK Health Research Authority guidance, formal Research Ethics Committee approval and individual informed consent were not required.

## Results

### Baseline characteristics

A total of 991 patients with newly diagnosed GD (mean age 48.1 ± 16.3 years; 82% women) were included (Table 1). At baseline, 27% were current smokers, 23% ex-smokers, and 50% nonsmokers. Among current smokers, median (IQR) cigarette consumption was 10/day (10-16). The median duration of ATD therapy was 13 (12-18) months, with median follow-up after ATD withdrawal of 31 (12-80) months (Table 1).

**Table 1:** Baseline characteristics of patients with newly diagnosed Graves' disease according to smoking status

Variable	All patients (*n* = 991)	Nonsmokers (*n* = 491)	Ex-smokers (*n* = 232)	Current smokers (*n* = 268)	*P*-value *^a^*
Age (years)	48.1 ± 16.3	46.2 ± 17.1	53.4 ± 15.6	46.8 ± 14.5	<.001
Female sex, *n* (%)	808 (81.5)	404 (82.3)	182 (78.8)	222 (82.5)	.47
BMI (kg/m²)	26.5 ± 6.1	26.5 ± 6.1	27.7 ± 5.9	25.3 ± 6.0	<.001
Graves' orbitopathy present, *n* (%)	152 (15.4)	65 (13.3)	29 (12.6)	58 (21.8)	.003
FT4 (pmol/L)	39.9 [28.6-60.0]	42.5 [30.2-62.3]	38.1 [26.7-58.9]	37.2 [27.2-57.9]	.03
FT3 (pmol/L)*^b^*	15.2 [9.6-26.1]	15.8 [10.1-26.0]	13.7 [8.9-24.9]	14.8 [9.5-27.6]	.16
TRAb (IU/L)	6.7 [3.7-14.3]	6.6 [3.7-13.7]	5.5 [3.0-13.2]	8.0 [4.4-16.4]	.002
TPOAb (IU/mL), (*n* = 792)	111 [20-321]	103 [17-254] (*n* = 393)	106 [19-338] (*n* = 192)	160 [31-399] (*n* = 207)	<.01
Tc-pertechnetate uptake (%), (*n* = 396)	5.4 [3.1-9.2]	6.4 [3.6-11.8] (*n* = 195)	5.5 [3.3-8.7] (*n* = 89)	3.5 [2.0-6.5] (*n* = 112)	<.001
Thyroid area (cm²), (*n* = 352)	24.5 ± 7.5	24.3 ± 7.4 (*n* = 176)	24.2 ± 5.6 (*n* = 78)	25.0 ± 9.0 (*n* = 98)	.74

Abbreviations: BMI, body mass index, calculated by dividing the weight in kilograms by the height in meters squared; FT3, free triiodothyronine; FT4, free thyroxine.

^*a*^ *P*-values from 1-way ANOVA or Kruskal–Wallis tests for continuous variables and χ² tests for categorical variables.

^*b*^Data were missing for 78 patients for FT3 levels. There was no data missing for the other variables unless otherwise stated.

Values are mean ± SD or median (IQR) unless otherwise specified. Bold values indicate statistically significant group differences (*P* < .05).

To convert FT4 from pmol/L to ng/dL multiply by 0.08. To convert FT3 from pmol/L to pg/dL multiply by 64.94.

### Smoking, TRAb and thyroid hormone levels

**Figure 1:**
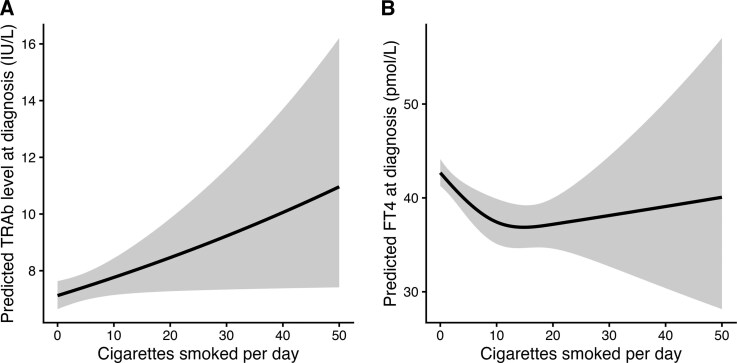
Relationship between cigarette consumption, TRAb, and FT4 levels. Predicted TRAb concentrations (IU/L) (A) and Free T4 (pmol/L), back-transformed from the log scale, are shown on the original scale, derived from a multivariable linear regression model with TRAb and FT4 as the dependent variables, respectively. The model was adjusted for age, sex, and BMI (A) and age, sex, BMI, and TRAb levels (B). The solid black line represents the adjusted mean prediction, and the shaded area denotes the 95% confidence interval. The relationship was approximately linear (*P* for nonlinearity = .20) for TRAb and nonlinear for FT4 (*P* for nonlinearity < .05). Confidence intervals widen at higher cigarette counts where data are sparse. To convert FT4 from pmol/L to ng/dL multiply by 0.08.

Median TRAb concentrations at diagnosis differed significantly by smoking status (*P* = .002): current smokers had the highest levels (8.0 IU/L [IQR 4.4-16.4]), followed by nonsmokers (6.6 [3.7-13.7]) and ex-smokers (5.5 [3.0-13.2]). In multivariable regression, each 10 cigarettes/day were associated with a 9% higher TRAb level (β = .087, *P* = .04), independent of age, sex, and BMI (Fig. 1A). The relationship was linear (*P* for nonlinearity = .20). Older age and higher BMI were associated with lower TRAb, while sex was not.

The number of cigarettes smoked per day was significantly associated with FT4 levels (*P* = .0015), showing a mild nonlinear relationship (*P* = .049): FT4 concentrations declined with increasing cigarette consumption up to approximately 10-15 cigarettes/day, then rose slightly at higher intensities (Fig. 1B). Free triiodothyronine levels showed no significant association (*P* = .095). At baseline, nonsmokers had slightly higher median FT4 (42.5 [30.2-62.3]) than current smokers (37.2 [27.2-57.9]; *P* = .03), but FT3 did not differ (*P* = .16).

### Smoking and Graves' orbitopathy

At diagnosis, 15.4% of patients had GO, most commonly among current smokers (21.8%) compared with ex-smokers (12.6%) and nonsmokers (13.3%; *P* < .001). After adjustment, current smoking remained an independent predictor of GO (OR 1.76; 95% CI 1.17-2.64; *P* = .007), while ex-smokers did not differ from nonsmokers (OR 0.98; *P* = .91).

**Figure 2:**
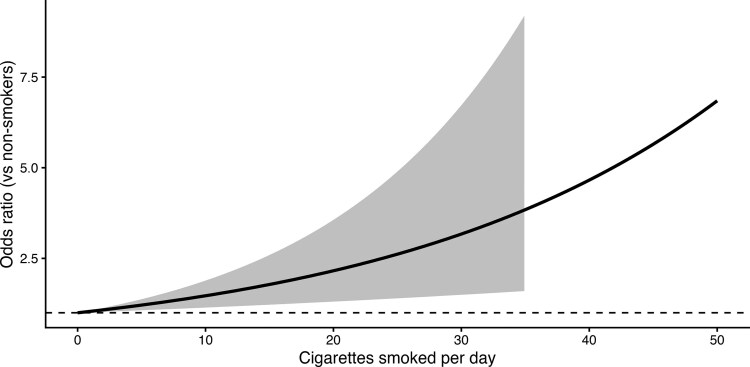
Risk of GO by daily number of cigarettes. Predicted odds ratios (solid black line) and 95% confidence intervals (shaded area) are shown for the association between the number of cigarettes smoked per day and the presence of GO at diagnosis, adjusted for age, sex, BMI, and TRAb levels. The dashed horizontal line represents the reference level (nonsmokers, OR = 1). Increasing cigarette consumption was associated with progressively higher odds of orbitopathy, with approximately 1.8-fold higher odds for a 20-cigarette/day smoker compared with a nonsmoker. Confidence intervals are truncated at higher smoking levels where data become sparse, reflecting fewer observations and reduced precision of estimates in that range.

Importantly, a continuous dose–response relationship was observed: each 10 cigarettes/day was associated with a 34% higher odds of GO (adjusted OR 1.34; 95% CI 1.10-1.79; *P* = .02), independent of TRAb, age, sex, and BMI (Fig. 2). Higher TRAb levels also predicted GO (OR 1.05 per unit; *P* < .001).

### Smoking and short- and long-term relapse risk

Among 663 patients completing ATD therapy with at least 12 months of follow-up, 154 (23.2%) relapsed. Relapse at 12 months was more frequent in current smokers (33%) than ex- (18%) or nonsmokers (20%; *P* = .001). In multivariable logistic regression, current smoking independently predicted relapse at 12 months (OR 1.92; 95% CI 1.26-2.91; *P* = .002), whereas ex-smokers were not at increased risk (OR 0.85; *P* = .52). Twelve-month relapse risk increased in a dose-dependent manner, with each 10 cigarettes/day associated with approximately 60% higher odds of relapse (*P* < .001). Higher baseline TRAb (*P* < .001) and older age (*P* = .05) were also associated with 12-month relapse.

**Figure 3:**
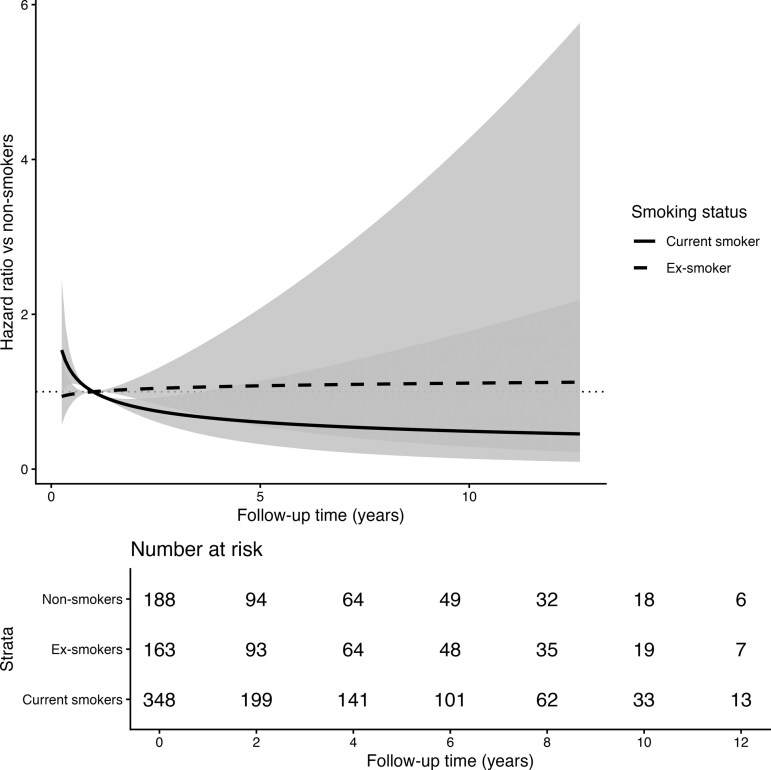
Time-varying hazard ratios for long-term risk of relapse by smoking status. Lines represent hazard ratios relative to nonsmokers from a Cox model with log-time interaction. Shaded areas show 95% confidence intervals, truncated at later follow-up due to limited data. The number at risk in each group is shown below the plot.

During extended follow-up (median 31 months; IQR 12-80), 43% relapsed after ATD withdrawal, with the highest frequency among current smokers (50%) compared with ex-smokers (38%) and nonsmokers (42%; *P* = .03). Time-dependent Cox models revealed that current smokers had a higher hazard of relapse early after ATD withdrawal (HR 1.24; 95% CI 1.05-1.47 at 6 months). This excess hazard attenuated progressively over time, such that by 12 months the relative hazard was equivalent by model definition, and thereby declined below unity. These later estimates likely reflect depletion of susceptible individuals among smokers rather than a protective (Fig. 3) (Table 2). Ex-smokers did not differ from nonsmokers at any time point. Baseline TRAb predicted long-term relapse (+2% per unit; *P* = .04). Cigarette number did not independently predict long-term relapse risk, with no significant association observed per 10 cigarettes/day (HR 1.03; *P* = .30).

**Table 2:** Time-varying relative hazard of long-term relapse in current and ex-smokers compared with nonsmokers

Follow-up (months)	HR (95% CI) for current vs nonsmokers	HR (95% CI) for ex- vs nonsmokers
6	1.24 (1.05-1.47)	0.97 (0.81-1.16)
12	1.00 (1.00-1.00)	1.00 (1.00-1.00)
24	0.81 (0.68-0.95)	1.03 (0.87-1.23)
36	0.71 (0.54-0.93)	1.05 (0.80-1.39)
60	0.61 (0.41-0.90)	1.08 (0.72-1.62)
120	0.49 (0.28-0.86)	1.11 (0.62-1.99)

Hazard ratios (HRs) represent time-specific effects estimated from time-dependent Cox proportional hazards models adjusted for age, sex, BMI, baseline TRAb levels, and duration of antithyroid drug therapy. The model was centered at 12 months, where the HR equals 1.0 by definition; values at later time points reflect the change in relative relapse hazard over time compared with nonsmokers at the same follow-up duration.

### Smoking, treatment duration, and TRAb dynamics

Current smokers remained on ATD therapy longer than non- or ex-smokers in Kaplan–Meier analysis (mean 19.4 vs 15.9 and 16.2 months, respectively; log-rank *P* = .01), although median durations were similar. After adjustment for age, sex, and baseline TRAb, smoking status was not independently associated with treatment duration (HR 0.88, 95% CI 0.73-1.04; *P* = .14), while higher baseline TRAb predicted longer therapy (HR 0.96 per unit increase; *P* < .001).

Among patients with TRAb data at cessation (n = 681), smokers maintained higher titers. In adjusted regression, current smoking (β = .10; 95% CI 0.03-0.17; *P* = .004), older age (*P* < .001), and higher baseline TRAb (*P* < .001) independently predicted TRAb at cessation, whereas treatment duration did not. Each 10 cigarettes/day was associated with higher log TRAb at ATD cessation (β = .16; 95% CI 0.08-0.25; *P* < .001) (or ∼17% higher TRAb at ATD cessation), confirming a dose-dependent delay in immunological remission among smokers.

### Smoking, TPOAb, symptoms, and technetium uptake

In multivariable linear regression, each 10 cigarettes/day showed no significant association with log-transformed TPOAb concentrations (β = .15, *P* = .10). Restricted cubic spline analysis likewise demonstrated no evidence of nonlinearity (*P* = .16), indicating that smoking intensity was not meaningfully associated with TPOAb titers. Median TPOAb concentrations differed significantly across smoking groups (Table 1), with current smokers showing higher titers (adjusted *P* = .005), while differences involving ex-smokers were not significant.

Smoking status was not associated with presenting hyperthyroid symptoms after adjustment for age, sex, BMI, and FT4 (*P* = .51).

Technetium-99 m pertechnetate uptake varied significantly (*P* < .001): nonsmokers had the highest uptake (6.4 [3.7-11.8] %), and current smokers the lowest (3.5 [2.0-6.6] %), while thyroidal area was similar (*P* = .70). Post hoc tests confirmed significantly reduced uptake in current smokers vs both non- (*P* < .001) and ex-smokers (*P* = .01).

### Mediation analysis

TRAb levels partially mediated the association between current smoking and relapse at 12 months (ACME effect = 0.009; 95% CI 0.000-0.021; *P* = .04), explaining 6.6% of the total effect. The direct effect remained significant (*P* = .002), indicating that smoking's impact on relapse was only partly TRAb-mediated. For long-term relapse, mediation by TRAb was negligible (*P* = .66), supporting largely TRAb-independent mechanisms.

## Discussion

In this large cohort of nearly 1000 patients with newly diagnosed GD, cigarette smoking was independently associated with higher TRAb concentrations at diagnosis, modestly lower FT4 levels showing a nonlinear dose–response pattern, reduced thyroidal technetium uptake, a greater prevalence of GO, and increased relapse risk following ATD therapy. Many associations demonstrated clear dose–response relationships, and ex-smokers consistently showed outcomes similar to nonsmokers, underscoring the reversibility of smoking-related effects. Collectively, these findings reinforce that active smoking adversely affects both the autoimmune and clinical course of GD, and that smoking cessation, or even reduction, may improve disease trajectory.

Evidence from other autoimmune diseases, including rheumatoid arthritis, Crohn's disease, multiple sclerosis, and psoriasis, shows that smoking worsens outcomes in a dose-dependent fashion and that cessation is associated with partial reversal of risk, even in the absence of formal cessation trials powered for disease endpoints (16). These parallels strengthen biological plausibility that modifying smoking behavior could meaningfully alter GD outcomes.

### Smoking, thyroid autoantibodies, thyroid function, and treatment duration

Current smokers exhibited significantly higher TRAb titers than ex- or nonsmokers, with approximately a 9% increase in TRAb for every 10 additional cigarettes smoked per day. This dose–response relationship is consistent with earlier evidence linking smoking with augmented thyroid autoimmunity and suggests that nicotine-related oxidative stress and immune activation amplify TRAb production (6, 17, 18). As observed previously, smokers also had higher TRAb levels at treatment cessation, independent of treatment duration, indicating slower immunological remission (19). Even moderate smoking can hinder immunologic recovery, highlighting potential benefits of smoking reduction as well as cessation (20). Nicotine-induced oxidative stress and altered T-cell activity may enhance autoantibody production, effects that appear partly reversible after cessation (21).

Thyroid peroxidase antibodies concentrations were also modestly higher in current smokers, although the association with cigarette intensity was weaker, suggesting differential susceptibility of antibody classes to smoking-related immune modulation.

A nonlinear relationship was identified between smoking intensity and FT4 concentrations, with slightly higher FT4 levels among light smokers and progressively lower FT4 at higher cigarette consumption. This pattern may reflect competing physiological effects: low-level nicotine exposure may transiently stimulate thyroid activity, whereas heavier smoking and thiocyanate accumulation inhibit iodide transport or hormone synthesis and release (20). These findings suggest that while smoking not only augments thyroid autoimmunity but may also impair thyroidal responsiveness to TSH receptor stimulation, potentially through smoking-induced hypoxia, oxidative stress, inhibition of iodide transport, or shifts in TRAb functional subclasses (1). Consistent with this, technetium uptake was significantly lower in smokers despite similar thyroid size, implying impaired thyroidal trapping.

Although smokers remained on ATD therapy longer in unadjusted analyses, this effect was explained by higher baseline and persistent TRAb levels rather than treatment decisions. These results highlight that smoking affects both immunological and metabolic determinants of thyroid function in GD.

### Smoking and Graves' orbitopathy

GO was present in 15% of patients, but nearly 1 in 5 current smokers had ocular involvement, approximately double the prevalence observed in nonsmokers. This association remained robust after adjustment for TRAb and other covariates, confirming smoking as an independent risk factor (22). Risk increased in a dose-dependent manner, with each additional cigarette per day conferring incremental odds of GO. These findings extend prior observations by demonstrating a continuous exposure–response relationship, suggesting that even partial smoking reduction may reduce risk. Proposed mechanisms include increased TSH receptor expression on orbital fibroblasts, hypoxia-induced adipogenesis, and local cytokine activation (23). Ex-smokers had GO rates similar to nonsmokers, highlighting reversibility and the importance of smoking modification as part of comprehensive GO prevention and management.

### Smoking and relapse risk

Smoking was associated with increased relapse following ATD withdrawal. Current smokers had nearly double the 12-month relapse rate of nonsmokers, and relapse risk rose approximately 5% per additional cigarette per day, indicating a strong dose–response effect. Over longer follow-up, the excess hazard declined, suggesting that smoking's detrimental impact is greatest early after treatment cessation. Ex-smokers again showed relapse rates comparable to nonsmokers, reinforcing the potential benefits of cessation. These findings expand on earlier, smaller studies and support the hypothesis that continued smoking sustains autoimmune activity or interferes with restoration of immune tolerance (5, 24-26).

### Mediation by TRAb levels

Mediation analysis showed that baseline TRAb explained only ∼7% of the excess short-term relapse risk in smokers, and none of the long-term effect, suggesting that smoking acts through both TRAb-dependent and TRAb-independent mechanisms. Broader immunomodulatory pathways, altered drug metabolism, or behavioral factors may contribute.

### Clinical implications

Smoking cessation should be an essential component of GD management, reducing the risk of GO and improving the probability of durable remission. Quantifying smoking exposure at diagnosis may improve prognostic assessment, as even moderate consumption carried measurable risk. As novel immunotherapies for GD emerge, with greater cost, complexity, and potential adverse effects, the simplicity, safety and broad health benefits of smoking cessation or reduction should not be overlooked. Incorporating structured cessation support into routine GD care may represent one of the most cost-effective strategies to improve outcomes.

### Strengths and limitations

Strengths include the large, real-world cohort, detailed phenotyping, and evaluation of both categorical and continuous smoking exposure. Limitations include reliance on self-reported smoking, absence of passive smoking data, incomplete information on smoking changes during follow-up, and potential residual confounding. Missing data were handled by complete-case analysis, which may introduce bias if data were not missing at random, although the proportion of missingness was small for most variables. Variability in assay platforms over time may have introduced minor measurement differences. Findings are most generalizable to similar secondary-care populations.

## Conclusions

Smoking is an independent, dose-dependent determinant of GD severity and prognosis. Ex-smokers revert toward baseline risk, highlighting reversibility. Smoking cessation—or even reduction—represents a simple, safe, and low-cost therapeutic target that should remain central to comprehensive GD management.

## Data Availability

Some or all datasets generated during and/or analyzed during the current study are not publicly available but are available from the corresponding author on reasonable request.
